# Association Between Iodine Nutritional Status and Adverse Pregnancy Outcomes in Beijing, China: a Single-Center Cohort Study

**DOI:** 10.1007/s12011-021-02887-9

**Published:** 2021-09-27

**Authors:** Xiaomei Zhang, Ning Yuan, Jianbin Sun, Xin Zhao, Jing Du, Min Nan, QiaoLing Zhang, Linong Ji

**Affiliations:** 1 Department of Endocrinology, Peking University International Hospital, Beijing, 102206 China; 2 Department of Endocrinology, Peking University People’s Hospital, Beijing, 100044 China

**Keywords:** Iodine, Thyroid antibodies, Pregnancy outcomes, Fetal growth parameters

## Abstract

Iodine is an essential trace element for humans and the main raw material for thyroid hormone synthesis. However, the association between iodine nutritional status and adverse pregnancy outcomes in different regions remains controversial. This single-center cohort study was focused on the association between iodine nutritional status and adverse pregnancy outcomes in Beijing, China. We enrolled 726 pregnant women who were registered at the Peking University International Hospital between February 2017 and December 2019. To analyze the association between iodine nutritional status variations and adverse pregnancy outcomes, this study cohort included 390 (53.72%) participants with iodine deficiency, 206 (28.37%) with an adequate iodine level, 103 (14.19%) with a more than adequate iodine level, and 27 (3.72%) with iodine excess, according to the urinary iodine (UI) status of pregnant women. After adjusting for age, body mass index, parity, and history of spontaneous abortion, we identified iodine deficiency as a risk factor for anti-thyroid peroxidase antibody (TPOAb) positivity [odds ratio (OR), 3.646; 95% confidence interval (95% CI), 1.658–8.017], anti-thyroglobulin antibody (TGAb) positivity (OR, 3.109; 95% CI, 1.465–6.599), and thyroid autoimmunity (OR, 2.885; 95% CI, 1.539–5.407). There was a non-linear relationship between UI and the concentrations of TPOAb and TGAb (*P*_non-linear_ < 0.05). Iodine deficiency during the first trimester is a risk factor for thyroid autoantibody positivity. The relationship between UI and the concentrations of TPOAb and TGAb follows a nearly U-shaped curve. Thus, physicians should critically consider the iodine nutritional status of pregnant women during the first trimester. Clinical Trials.gov Identifier: NCT02966405

**Supplementary Information:**

The online version contains supplementary material available at 10.1007/s12011-021-02887-9.

## Introduction

Iodine, an essential trace element, is absorbed by the thyroid gland to synthesize triiodothyronine (T3) and thyroxine (T4), which are vital for normal human development, cell metabolism, and the development of the fetal brain and other parts of the nervous system. In the first trimester of pregnancy, the mother-to-be provides the iodine needed for producing the thyroid hormones in the fetus. Therefore, fetal growth and brain development critically depend on adequate maternal iodine intake during pregnancy. Pregnant women are at a high risk of iodine deficiency due to increased urinary iodine (UI) clearance and fetal iodine requirements. the Chinese government has implemented the Universal Salt Iodization (USI) policy, which has substantially contributed to the elimination of iodine deficiency diseases in China. In 2007, the World Health Organization (WHO), the United Nations Children’s Fund (UNICEF), and the Iodine Global Network (IGN, formerly known as the International Council for the Control of Iodine Deficiency Disorders) proposed an evaluation criteria for the iodine nutritional status of pregnant women [1]. A median UI concentration of 150–249 µg/L indicates an adequate iodine nutritional status in pregnant women. Previous studies observed an association between iodine deficiency and adverse pregnancy outcomes. Specifically, there is the finding that iodine deficiency during pregnancy is associated with hypothyroidism, subclinical hypothyroidism (SCH), and hypothyroxinemia, which can lead to spontaneous abortion, premature delivery, premature rupture of membrane (PROM), and other adverse pregnancy outcomes [2]. Other reports show that iodine deficiency during pregnancy can directly cause premature birth, fetal growth restriction, and nervous system development abnormalities [3, 4]. Although iodine excess has low morbidity during pregnancy, it can also cause adverse pregnancy outcomes [5]. In contrast, some studies reported that an abnormal iodine status is not associated with an adverse pregnancy outcome [6, 7]. However, iodine is essential for fetal growth and brain development. A prospective cohort study showed that both maternal iodine insufficiency and excess in the first trimester of pregnancy adversely affected fetal growth [8]. A separate meta-analysis of the association between UI and pregnancy outcomes in euthyroid pregnant women did not detect an association between UI and fetal birth indicators [9]. In conclusion, there is still controversy about the association between the iodine nutritional status and adverse pregnancy outcomes. This study was focused on China’s capital Beijing, which is an iodine-sufficient region. Our primary objective was to explore the association between the iodine nutritional status and adverse pregnancy outcomes. The secondary objectives included an assessment of the iodine nutritional status of pregnant women in this region, as well as the association between UI and thyroid function, thyroid autoantibodies, and fetal length and weight.

## Methods

### Participants

This single-center cohort study included 726 pregnant women who were admitted to the Department of Obstetrics, Gynecology, and Endocrinology at the Peking University International Hospital between February 2017 and December 2019. Eligibility criteria included single birth, the first trimester of pregnancy (4–8 weeks), long-term residence (≥ 5 years) in the Beijing region, and voluntary participation in the study. Exclusion criteria included hereditary diseases, tumors, autoimmune diseases (such as systemic lupus erythematosus, Sjogren’s syndrome, or antiphospholipid antibody syndrome), heart disease, liver disease, kidney disease, or chronic hypertension, as well as medications that may affect thyroid function.

### Ethics Approval

This study was approved by the Ethics Committee of the Peking University International Hospital [2017–021(BMR)]. Written informed consent was obtained from each pregnant woman.

### Procedures

All enrolled participants completed a questionnaire about previous medical history, family history, fertility history, and residence status. The last menstrual period, height, weight, heart rate, and blood pressure of the participants were recorded. Body mass index (BMI) was calculated as the participant’s weight in kilograms (kg) divided by her height in meters squared (m^2^). The monitored blood biochemical indexes included serum blood glucose (GS), glycosylated hemoglobin (HbA1c), low-density lipoprotein cholesterol (LDL-C), uric acid (UA), homocysteine (Hcy), and ferritin, along with thyroid function parameters, including thyroid stimulating hormone, (TSH) free thyroxine (FT4), anti-thyroglobulin antibody (TGAb), anti-thyroid peroxidase antibody (TPOAb), and UI. The pregnant women were followed up until delivery. The mode of birth, delivery time, and neonatal gender, weight, and height were recorded along with the pregnancy outcomes.

### Definitions

#### Iodine Nutritional Status Criteria

To assess the population iodine nutritional status based on the median UI concentration, we used the criteria proposed by the WHO, UNICEF, and IGN in 2007 [1], as shown in Table 1.

**Table 1:** Criteria for iodine nutritional status of pregnant women

Iodine nutrition status of pregnant women	UI (μg/L)
Iodine deficiency	< 150
Iodine adequate	150–249
More than iodine adequate	250–499
Iodine excess	≥ 500

*UI*, urinary iodine

#### Definition of Thyroid Disease During Pregnancy

Based on the specific reference range of thyroid function during pregnancy at the Peking University International Hospital, thyroid diseases included hyperthyroidism, hypothyroidism, SCH, hypothyroxinemia, TPOAb and TGAb positivity, and thyroid autoimmunity (TAI) (Supplementary Table 1).

#### Definition of Pregnancy Outcomes

Pregnancy and fetal outcomes included gestational diabetes mellitus (GDM), spontaneous abortion (PROM), hypertensive disease during pregnancy (HDP), preterm delivery, fetal distress, low birth weight, macrosomia, and small for gestational age (SGA). These definitions of adverse pregnancy outcomes were consistent with previous studies [10].

### Statistical Analysis

All data were statistically analyzed using SPSS version 17.0 (SPSS Inc., Chicago, IL, USA). The mean ± standard deviation (X ± SD) was used for the statistical analysis of normally distributed datasets, and the *t* test was applied for assessing the statistical inference. The median and interquartile range (IQR) were used for the statistical analysis of datasets with a non-normal distribution, and the Mann–Whitney *U* test was performed for assessing the statistical inference. Enumeration datasets were statistically analyzed by calculating the constituent ratio or rate, and the statistical inference was assessed by applying the *χ*^2^ test or Fisher’s exact probability method. The multivariate logistic regression model was used to evaluate the relationship between different iodine nutritional status levels and adverse pregnancy outcomes. The non-linear relationships between UI and thyroid function, TGAb, TPOAb, birth weight, and height of neonates were analyzed by restricted cubic spline regression using R language from the Central R Archive Network version 3.5.2 and R Studio 1.3 (segmented, splines, ggplot2, Hmisc, and rms packages). *P* < 0.05 was considered statistically significant.

## Results

A total of 726 pregnant women in the first trimester were enrolled in this study according to the inclusion and exclusion criteria. The enrolled participants included 390 (53.72%) pregnant women with iodine deficiency, 206 (28.37%) with an adequate iodine level, 103 (14.19%) with a more than adequate iodine level, and 27 (3.72%) with iodine excess, according to the UI status of pregnant women [1]. Thus, based on the iodine nutritional status in the first trimester of pregnancy, we divided the study participants into three groups: the iodine deficiency group (*n* = 390), the iodine-adequate group (*n* = 206), and the more than iodine-adequate plus iodine excess group (*n* = 130). Specifically, the iodine deficiency, the iodine-adequate, and the more than iodine-adequate plus iodine excess groups had median UI concentrations of 86 μg/L, 187 μg/L, and 336 μg/L, along with median UI to creatinine (Cr) ratios (UI/Cr) of 59.64 μg/g, 113.59 μg/g, and 221.09 μg/g, respectively (Table 2). There were significant differences among the three groups of pregnant women for the UI and UI/Cr values (*P* < 0.001), whereas no group-dependent differences were observed for the other parameters, including age, BMI, birth order, history of spontaneous abortion, HbA1c, GS, TSH, FT4, LDL-C, UA, Hcy, and ferritin.

**Table 2:** Subject’s characteristics of pregnant women in different iodine nutritional status groups

	Iodine deficiency group(*n* = 390)	Iodine-adequate group(*n* = 206)	More than iodine- adequate plus iodine excess group (*n* = 130)	Statistics	*P* value
Maternal age (years)	30 (28, 33)	30 (28, 33)	30 (28, 32)	1.101	0.577
BMI (kg/m^2^)	21.22 (18.59, 23.34)	22.60 (19.21, 25.68)	19.38 (17.36, 21.77)	1.499	0.473
Primipara (%)	250 (64.1%)	137 (66.5%)	95 (73.1%)	3.521	0.172
History of spontaneous abortion (%)	19 (4.9%)	11 (5.3%)	8 (6.2%)	0.330	0.848
HbA1c (%)	5.20 (5.09, 5.40)	5.20 (5.09, 5.40)	5.20 (5.00, 5.40)	2.069	0.355
GS (mmol/L)	4.80 (4.60, 5.01)	4.80 (4.55, 5.00)	4.82 (4.50, 5.10)	0.471	0.790
TSH (uIU/ml)	1.64 (0.85, 2.62)	1.79 (1.15, 2.55)	1.76 (1.06, 2.67)	2.429	0.297
FT4 (pmol/L)	17.30 (15.90, 18.90)	17.30 (16.00, 19.30)	17.70 (16.20, 19.50)	1.857	0.395
LDL (mmol/L)	2.04 (1.71, 2.38)	1.98 (1.66, 2.35)	2.03 (1.66, 2.50)	0.948	0.623
UA (μmol/L)	216 (184, 247)	216 (187, 249)	208 (181, 238)	1.326	0.515
Hcy (μmol/L)	6.60 (5.80, 7.40)	6.30 (5.80, 7.17)	6.3 (5.60, 7.20)	4.011	0.135
Ferritin (ng/ml)	55.4 (36.1, 83.5)	53.9 (36.0, 84.2)	57.9 (39.8, 95.1)	3.414	0.181
UI (μg/L)	86 (50, 121)	187 (167, 208)^a^	336 (285, 462)^a,b^	591.911	0.000^*^
UI/ urine Cr (μg/g)	59.64 (89.14, 138.80)	113.59 (80.92, 168.18)^a^	221.09 (145.25, 295.08)^a,b^	161.587	0.000^*^

^a^Compared with the iodine deficiency group; ^b^Compared with the iodine - adequate group; *P < 0.05

Statistics: Maternal age, BMI, HbA1c, GS, TSH, FT4, LDL, UA, Hcy, Ferritin UI, and UI/urine Cr for Mann–Whitney *U* test; parity, and history of spontaneous abortion for chi-square test or Fisher test. Continuous data are expressed as median (interquartile range). *BMI*, body-mass index; *GS*, blood glucose; *HbA1c*, glycated hemoglobin; *TSH*, thyroid stimulating hormone; *FT4*, free thyroxine; *LDL*, low density lipoprotein cholesterol; *UA*, uric acid; *Hcy*, homocysteine; *UI*, urine iodine; *Cr*, creatinine.

As shown in Table 3, the rates of TPOAb positivity, TGAb positivity, and TAI differed among the three study groups. Specifically, the iodine deficiency group had significantly higher rates of TPOAb positivity and TAI than the other two groups (TPOAb positivity: 19.2% vs. 11.7% and 10%; TAI: 25.9% vs. 16.5% and 14.6%; *P* < 0.05). The TGAb positivity rates significantly differed among the study groups (*P* < 0.05). It was the highest in the iodine deficiency group, lower in the more than iodine-adequate plus iodine excess group, and the lowest in the iodine-adequate group. The birth height of the fetus significantly varied between the iodine deficiency group and the more than iodine-adequate plus iodine excess group (*P* < 0.05). The iodine deficiency group had higher incidences of SCH, PROM, macrosomia, and SGA than the iodine-adequate group and the more than iodine-adequate plus iodine excess group, but these differences were not statistically significant. The incidences of GDM, spontaneous abortion, HDP, preterm delivery, fetal distress, birth weight, height, and gestational age barely varied among the three groups.

**Table 3:** The pregnancy and fetal outcomes in different iodine nutritional status groups

	Iodine deficiency group(*n* = 390)	Iodine-adequate group(*n* = 206)	More than iodine-adequate plus iodine excess group (*n* = 130)	Statistics	*P* value
GDM (%)	13 (3.3%)	13 (6.3%)	6 (4.6%)	2.852	0.240
SCH(%)	23 (5.9%)	12 (5.8%)	5 (3.8%)	0.843	0.656
Hypothyroxidemia	8 (2.1%)	5 (2.4%)	2 (1.5%)	0.312	0.856
TPOAb positivity(%)	75 (19.2%)	24 (11.7%)^a^	13 (10%)^a^	9.511	0.009^*^
TGAb positivity(%)	75 (19.2%)	23 (11.3%)^a^	19 (14.6%)^a,b^	6.537	0.038^*^
TAI	101 (25.9%)	34 (16.5%)^a^	19 (14.6%)^a^	11.239	0.004^*^
Abortion (%)	14 (3.6%)	12 (5.8%)	11 (8.5%)	5.100	0.078
PROM (%)	66 (16.9%)	29 (14.1%)	16 (12.3%)	1.930	0.381
HDP (%)	10 (2.6%)	5 (2.4%)	4 (3.1%)	0.141	0.932
Preterm birth (%)	15 (3.8%)	10 (4.9%)	8 (6.2%)	1.260	0.533
Fetal distress (%)	16 (4.1%)	12 (5.8%)	8 (6.2%)	1.329	0.515
Low birth weight (%)	9 (2.3%)	9 (4.4%)	6 (4.6%)	2.641	0.267
Macrosomia (%)	25 (6.4%)	12 (5.8%)	7 (5.4%)	0.208	0.901
SGA (%)	3 (0.8%)	1 (0.5%)	NA	0.163	0.687
Infant					
Birth height(cm)	50 (49, 52)	50 (49, 51)	50 (49, 51)^a^	8.882	0.012^*^
Birth weight (Kg)	3.41 (3.07, 3.68)	3.26 (3.00, 3.60)	3.29 (3.03, 3.67)	5.383	0.068
Gestational age (weeks)	39 (38, 40)	39 (38, 40)	39 (38, 40)	3.853	0.146

^a^Compared with the iodine deficiency group; ^b^Compared with the iodine - adequate group; *P < 0.05

*GDM*, gestational diabetes mellitus; *SCH*, subclinical hypothyroidism; *TPOAb*, anti-thyroid peroxidase antibody; *TGAb*, anti-thyroglobulin antibody; *TAI*, thyroid autoimmunity; *PROM*, premature rupture of membranes; *HDP*, hypertensive disease during pregnancy; *SGA*, small for gestational age

**Fig. 1:**
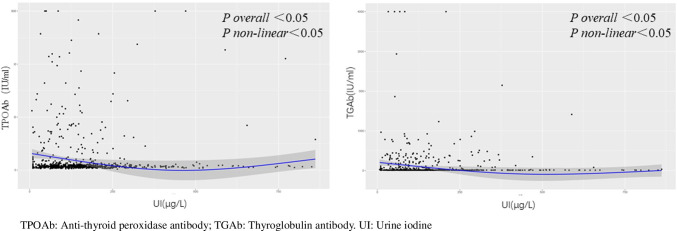
Restricted cubic spline regression analysis of urinary iodine and TPOAb and TGAb concentrations

**Fig. 2:**
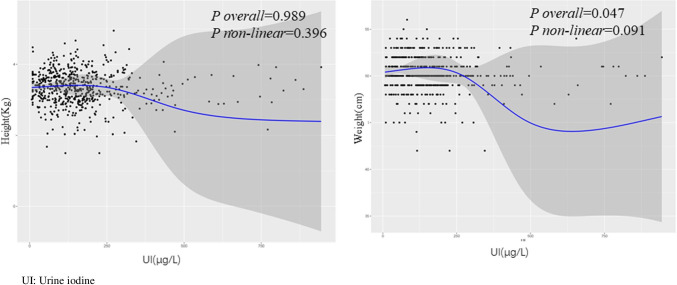
Restricted cubic spline regression analysis of urinary iodine and neonatal birth weight and height

As shown in Fig. 1, the graphical presentation of the relationship between UI and the concentrations of TPOAb and TGAb generated a U-shaped curve, and the restricted cubic spline regression analysis detected a non-linear relationship between UI and the two thyroid autoantibody concentrations (*P*_overall_ < 0.05, *P*_non-linear_ < 0.05). However, there was no non-linear relationship between UI and the birth weight and height of the newborn, based on the restricted cubic spline regression analysis (Fig. 2).

As shown in Table 4, iodine deficiency was a risk factor for TPOAb positivity [odds ratio (OR), 3.646; 95% confidence interval (95% CI), 1.658–8.017], TGAb positivity (OR, 3.109; 95% CI, 1.465–6.599), and TAI (OR, 2.885; 95% CI, 1.539–5.407) after adjustment for age, BMI, parity, and history of spontaneous abortion. However, there was no correlation between the iodine nutritional status and adverse pregnancy outcomes, including GDM, SCH, hypothyroxinemia, spontaneous abortion, PROM, HDP, preterm delivery, fetal distress, low birth weight, macrosomia, and SGA.

**Table 4:** Multivariate logistic regression analysis of the influence of different iodine nutrition status on the adverse pregnant outcomes

Pregnancy and fetal outcomes	Iodine sufficient	Iodine-adequate		More than iodine- adequate plus iodine excess	
	Ref	95% CI	*P* value	95% CI	*P* value
GDM	1	0.868 (0.253, 2.978)	0.821	1.138 (0.239–5.417)	0.871
SCH	1	0.791 (0.290, 2.157)	0.647	0.604 (0.150, 2.438)	0.479
Hypothyroxidemia	1	3.648 (0.439–30.322)	0.231	3.314 (0.284, 38.680)	0.339
TPOAb positivity	1	3.646 (1.658–8.017)	0.001^*^	1.237 (0.428, 3.572)	0.694
TGAb positivity	1	3.109 (1.465–6.599)	0.003^*^	2.055 (0.823–5.133)	0.123
TAI	1	2.885 (1.539–5.407)	0.001^*^	1.528 (0.686–3.404)	0.299
Abortion	1	1.934 (0.214–17.516)	0.557	4.844 (0.492–47.687)	0.176
PROM (%)	1	1.292 (0.736–2.268)	0.372	0.979 (0.471–2.032)	0.954
HDP (%)	1	1.205 (0.366–3.964)	0.759	1.600 (0.392–6.526)	0.513
Preterm birth	1	0.627 (0.239–1.648)	0.344	1.397 (0.497–3.927)	0.526
Fetal distress (%)	1	0.845 (0.331–2.160)	0.725	0.847 (0.263–2.729)	0.780
Low birth weight	1	1.386 (0.384–5.002)	0.618	0.951 (0.807, 1.120)	0.545
Macrosomia	1	0.931 (0.863–2.098)	0.863	0.401 (0.106–1.524)	0.180
SGA	1	1.203 (0.214–7.758)	0.834		

Adjusted for age, BMI, Parity, and history of spontaneous abortion

^*^P < 0.05

*GDM*, gestational diabetes mellitus; *SCH*, subclinical hypothyroidism; *TPOAb*, anti-thyroid peroxidase antibody; *TGAb*, anti-thyroglobulin antibody; *TAI*, thyroid autoimmunity; *PROM*, premature rupture of membranes; *HDP*, hypertensive disease during pregnancy; *SGA*, small for gestational age

## Discussion

The mother-to-be provides the iodine needed for fetal growth and development during the first trimester of pregnancy. Therefore, the maternal iodine nutritional status is not only related to the health of the pregnant woman but also directly affects the development of the brain and other parts of the nervous system in the fetus. This study mainly explored the association between the iodine nutritional status and adverse pregnancy outcomes. In our study population, the proportions of women with iodine deficiency, adequate iodine levels, more than adequate iodine levels, and iodine excess in the first trimester of pregnancy were 53.72%, 28.37%, 14.19%, and 3.72%, respectively. Thus, the common abnormal iodine nutritional status was iodine deficiency, followed by more than adequate iodine levels and iodine excess. This was consistent with some previous Chinese studies. A survey conducted in Dalian and Shenyang, China, found that the proportions of pregnant women with iodine deficiency, adequate iodine levels, more than adequate iodine levels, and iodine excess were 48.2%, 34.2%, 14.5%, and 3.2%, respectively [11]. In another survey conducted in the Zhejiang province, China, 61.7% and 2% of the pregnant women had iodine deficiency and iodine excess, respectively, while 20.9% had sufficient iodine levels [12]. The Chinese capital Beijing is an iodine-sufficient region that has implemented the USI policy. However, iodine deficiency was a common iodine abnormality in this study. Moreover, our study and previous reports suggested that iodine deficiency was the main abnormality of the iodine nutritional status during pregnancy in China. However, the iodine nutritional status in some other countries is different from the results of this study. In a cohort study in the USA, the iodine deficiency and iodine excess rates were 23% and < 1%, respectively [13]. A study about the iodine nutritional status in Israel found that 85% of the pregnant women had a UI level below the suitable range of 150–249 μg/L, suggesting that a national salt iodization policy should be urgently implemented for pregnant women [14]. The iodine nutritional status in different countries varies by region and depends on the iodine deficiency prevention policies. Iodine deficiency is common in pregnant women and should be critically considered in clinical practice.

In this study, the incidence of TPOAb positivity, TGAb positivity, and TAI varied among the different iodine nutritional status groups. After adjusting for age, BMI, parity, and history of spontaneous abortion, the multivariate logistic regression analysis showed that iodine deficiency was a risk factor for thyroid autoantibody positivity, but an adequate iodine level was not. The graphical presentation of the relationship between UI and the TPOAb and TGAb concentrations generated a U-shaped curve. Restricted cubic spline regression analysis detected a non-linear relationship between UI and the two thyroid autoantibodies (*P*_non-linear_ < 0.05), which was similar to previous studies. Shi X et al. found that iodine deficiency (UI < 100 μg/L) was associated with TPOAb and TGAb positivity in pregnant women [11]. A Swedish study on the UI/Cr ratio and thyroid autoantibodies in pregnant women showed a high rate of TPOAb positivity in pregnant women with UI/Cr < 150 μg/g (OR, 1.84; 95% CI, 1.07–3.20) [15]. A study of 7073 women in the first trimester of pregnancy in an iodine-sufficient region found that iodine deficiency (UI < 100 μg/L) was associated with TPOAb positivity [adjusted odds ratio (aOR), 1.64; 95% CI, 1.9–2.08] and TGAb positivity (aOR, 1.44; 95% CI, 1.16–1.80) [16]. Our single-center cohort study in Beijing, China, identified iodine deficiency as a risk factor for thyroid autoantibody positivity during pregnancy. Although there were differences between this study and previous studies in terms of region, iodine deficiency prevention policy, and sample size, the overall results suggested that iodine deficiency was associated with TPOAb and/or TGAb positivity. However, some studies did not detect a significant correlation between iodine deficiency and thyroid autoantibody positivity during pregnancy. A Norwegian study showed that TPOAb positivity was associated with a U-shaped curve for iodine intake in pregnant women with mild-to-moderate iodine deficiency. However, UI was not associated with TPOAb positivity [17]. Excess iodine can cause increased thyroglobulin iodization and stimulate the production of oxygen free radicals, which promote the metastasis of inflammatory cells, activate thyroid cell apoptosis, and lead to thyroiditis. This study and some previous reports did not show any association between iodine excess and thyroid autoantibody positivity during pregnancy. However, we found that the relationship between UI and the concentrations of TPOAb and TGAb followed a U-shaped curve. More studies are needed to investigate the relationship between excess iodine and thyroid autoantibodies. The influence of an abnormal iodine status on thyroid disease in pregnant women varies with race, region, time of inclusion, and thyroid autoantibody diagnostic criteria.

The mechanism underlying the effect of iodine deficiency on TAI during pregnancy is not very clear. It could be either a direct iodine effect on immune effector cells or a secondary response to a metabolic and/or toxic effect of iodine on thyroid tissue [18]. One study reported an association between iodine deficiency and enhanced proinflammatory activity [19]. Animal experiments showed that iodine had a direct effect on the immune cell or lymphocyte microenvironment and caused an increase in the number of inhibitory T cells in mice, leading to immune response [20]. Moreover, iodine deficiency can cause thyroid dysfunction. Low iodine intake can shift thyroid hormone utilization from T4 to the more biologically active T3 by upregulating peripheral type II deiodinase and increasing thyroid secretion of T3 [21, 22].

In this study, after adjusting for age, BMI, parity, and history of spontaneous abortion, multivariate logistic regression analysis showed that an abnormal iodine nutritional status was not a risk factor for GDM, spontaneous abortion, PROM, HDP, premature birth, fetal distress, low birth weight, macrosomia, and SGA. These observations were similar to the results of previous studies. A population-based prospective cohort study found that iodine deficiency, even moderate-to-severe iodine deficiency, did not increase the risk of abortion [7]. A study of maternal UI and pregnancy outcomes in three British cities found that the iodine nutritional status was not associated with birth weight and natural preterm delivery [6]. A meta-analysis of UI in pregnant women with normal thyroid function and pregnancy outcomes showed that the iodine nutritional status was not associated with preterm delivery, low birth weight, HDP, and neonatal birth characteristics [9]. In this study, we also included adverse pregnancy outcomes, such as GDM, PROM, fetal distress, macrosomia, and SGA, in addition to abortion, preterm delivery, HDP, and SGA. No association was found between the iodine nutritional status in pregnant women and these adverse pregnancy outcomes. However, some studies reported an association between abnormal iodine status and adverse pregnancy outcomes. A British birth cohort study found that maternal iodine deficiency was associated with lower levels of birth weight and an increased risk of SGA [4]. In another prospective cohort study, UI ≥ 250 μg/L was identified as an independent risk factor for GDM and HDP [23]. These variations may differ from the iodine nutritional status of other regions and the general status of enrolled pregnant women. Overall, researchers are increasingly concerned about the effect of the iodine nutritional status on other adverse pregnancy outcomes besides thyroid disease during pregnancy.

Adequate iodine intake in the first trimester of pregnancy is essential for fetal growth and brain development. Iodine placental transport to the embryo-fetus, and maternal iodine status regulates fetal growth by influencing placental weight [24]. An Argentina study [25] showed that pregnant women with iodine insufficiency are often associated with low placental weight, and there is a correlation between placental weight and head perimeter. However, there are few studies examining the effect of the iodine nutritional status in pregnant women on fetal growth. This study not only analyzed the association between UI and adverse pregnancy outcomes but also explored the association between UI and fetal birth indicators. In this study, the correlation analysis between UI and birth weight and height revealed no non-linear relationship, which was consistent with the results of some previous studies. A meta-analysis involving the effect of UI on fetal growth indicators showed no significant linear or non-linear relationship between the UI concentration in pregnant women and fetal growth parameters at birth, including weight, height, and head circumference [24]. A separate meta-analysis of the association between UI and pregnancy outcomes in euthyroid pregnant women did not detect an association between UI and birth weight, head circumference, Apgar score, and gestational age at birth [9]. In this meta-analysis, only euthyroid pregnant women were included, and the effects of thyroid hormones on fetal growth and development were excluded. There was no association between UI and fetal birth indicators. However, some studies found that UI < 1.0 mg/L was significantly positively correlated with birth weight, height, and head circumference of male fetuses [26]. Meanwhile, a prospective cohort study in Wuhan, China, found a negative association between iodine deficiency in pregnant women and fetal femoral length in the second and third trimesters. Longitudinal analysis showed that an abnormal iodine status in pregnant women was negatively correlated with fetal growth and development, which mainly included head circumference, femur length, and body weight [8]. Our study included weight and height as birth indicators but did not monitor other indicators, such as head circumference and femur length. Moreover, the sample size of our study was small. Therefore, more studies are needed to further clarify the effect of the iodine nutritional status on fetal birth indicators.

One limitation of this study was that the iodine intake of pregnant women was not recorded. However, Beijing has implemented the USI policy, and non-iodized salt needs to be purchased specifically. Typically, patients with a history of thyroid disease take iodized salt; however, the exclusion criteria for this study excluded pregnant women who took drugs affecting thyroid function. Another limitation was that this study was a single-center cohort study. However, the iodine nutritional status varies from region to region and depends on the policy to prevent iodine deficiency. This study may provide some evidence for the association between iodine nutrition and adverse pregnancy outcomes in pregnant women in the study region. Finally, the sample size of this study was limited, and only iodine deficiency was identified as a risk factor for thyroid disease in the first trimester of pregnancy. Increasing the number of study participants may identify more adverse outcomes during pregnancy.

## Conclusions

Iodine deficiency during the first trimester of pregnancy is a risk factor for thyroid autoantibodies positivity during pregnancy. The relationship between UI and the concentrations of TPOAb and TGAb follows a nearly U-shaped curve. In clinical practice, more attention should be paid to the association between the iodine nutritional status of pregnant women and adverse pregnancy outcomes.

## Supplementary Information

Below is the link to the electronic supplementary material.

Supplementary file1 (DOCX 16.5 KB)

## Data Availability

The datasets used and/or analyzed during the current study are available from the corresponding author on reasonable request.
