# Supplementary material for: Association Between Iodine Nutritional Status and Adverse Pregnancy Outcomes in Beijing, China: a Single-Center Cohort Study
Source: Biol Trace Elem Res. 2021 Sep 27;200(6):2620–8. doi: 10.1007/s12011-021-02887-9 (PMC9132840; doi:10.1007/s12011-021-02887-9)
Supplement: Supplementary file 1 — Supplementary file1 (DOCX 16.5 KB) [file 12011_2021_2887_MOESM1_ESM.docx]

Article title: Correlation between iodine nutritional status and adverse pregnancy outcomes in Beijing, China: A single-center cohort study

Journal name: Biological Trace Element Research

Xiaomei Zhang^1#^, Ning Yuan^1#^, Jianbin Sun^1^, Xin Zhao^1^, Jing Du^1^, Min Nan^1^, QiaoLing Zhang^1^, Linong Ji^2^*.

^1^Department of Endocrinology, Peking University International Hospital, Beijing 102206, China.

^2^Department of Endocrinology, Peking University People’s Hospital, Beijing 100044, China

*Corresponding author: Linong Ji, Email: JILINONG@pkuih.edu.cn; jiln@bjmu.edu.cn.

^#^These authors contributed equally to this work and should be considered co-first authors.

Supply table1 Definition of thyroid disease during pregnancy

|  | TSH (uIU/mL) | FT4 (pmol/L) | TPOAb and TGAb |
| --- | --- | --- | --- |
| Hyperthyroidism | <0.12 | >23.55 |  |
| Subclinical hyperthyroidism | <0.12 | 13.36-23.55 |  |
| Hypothyroidism | >4.16 | <13.36 |  |
| SCH | ＞4.16 | 13.36-23.55 |  |
| Hypothyroxidemia | 0.12-4.16 | <13.36 |  |
| TPOAb positivity |  |  | TPOAb>34 IU／mL |
| TGAb positivity |  |  | TGAb>115 IU／mL |
| TAI |  |  | TPOAb>34 IU／mL or TGAb>115 IU／mL |

TSH: thyrotropin; FT4: free thyroxine; SCH: subclinical hypothyroidism; TPOAb: anti-thyroid peroxidase antibody; TgAb: Thyroglobulin antibody; TAI: Thyroid autoimmunity.
